# Vitamin B_12_ deficiency and its impact on healthcare: a population-level analysis and call for action

**DOI:** 10.3389/fnut.2025.1701661

**Published:** 2026-01-05

**Authors:** Leonardo P. de Carvalho, Nelson Akamine, Marcelo S. Di Pietro, Carolina Nunes França, Rodrigo Oliveira, Renato D. Lopes

**Affiliations:** 1 Escola Paulista de Medicina, Universidade Federal de São Paulo, São Paulo, Brazil; 2 BCRI, Brazilian Clinical Research Institute, São Paulo, Brazil; 3 Health Sciences Post Graduation Program, Santo Amaro University, São Paulo, Brazil; 4 Duke Clinical Research Institute, Duke University Medical Center, Durham, NC, United States

**Keywords:** vitamin B_12_, health care, hospitalization, public health burden, clinical diseases

## Abstract

**Introduction:**

Vitamin B_12_ deficiency is a growing health concern, affecting millions worldwide. This study aims to evaluate the pattern of vitamin B_12_ deficiency in a large healthcare system and assess its impact on hospitalizations and associated diseases.

**Methods:**

Vitamin B_12_ test results were retrospectively collected from administrative datasets of Brazil’s public and private healthcare systems between 2016 and 2023. Data from health campaigns measuring vitamin B_12_ levels across the country were used to calculate the percentage of abnormal test results. The Mann-Kendall test was applied to assess trends in hospitalizations over time, while the Pearson correlation test was used to evaluate the association between vitamin B_12_ deficiency and other hospitalization causes.

**Results:**

A total of 84 million vitamin B_12_ measurements were analyzed, revealing a 12-fold increase in tests performed, rising from two million to 25 million over the study period. Of these, 35 million measurements were from the public and 47·8 million from private healthcare system. The trend of hospitalizations associated with vitamin B_12_ deficiency increased by 32% over the study period (*p* < 0·05). Hospitalizations associated with vitamin B_12_ deficiency were correlated with other B vitamin deficiencies (B_1_ and B_6_), as well as hospitalizations due to cardiovascular, hematological, neurological, psychological, and gastrointestinal diseases.

**Discussion:**

This population-based study highlights a progressive increase in vitamin B_12_ deficiency-related hospitalizations, suggesting a previously underrecognized public health burden. Additionally, these findings underscore the correlation between vitamin B_12_ deficiency, other B vitamin deficiencies, and relevant clinical diseases.

## 1 Introduction

Vitamin B12 deficiency has emerged as a global health concern with region-specific determinants and outcomes (1). In Brazil, this issue deserves particular attention due to marked socioeconomic disparities, evolving dietary habits, and the coexistence of both undernutrition and chronic disease burdens. Some of these reasons include changing dietary patterns due to the increased adoption of plant-based diets (2), an aging population (3), and health conditions such as gastrointestinal disorders and long COVID-19 syndrome (4, 5), which can affect nutritional status. Additionally, social inequality and food insecurity—especially in underdeveloped and developing countries like Brazil—also contribute to these challenges (6, 7).

Vitamin B_12_ is a water-soluble vitamin primarily obtained from animal products such as red meat, dairy, and eggs. Intrinsic factor, a glycoprotein produced by parietal cells in the stomach, is essential for vitamin B_12_ absorption in the terminal ileum. The recommended daily intake for adults is 2.4 micrograms (8). Since the human body cannot synthesize vitamin B_12_, it must be obtained through food or supplements.

Once absorbed, the body utilizes vitamin B_12_ to produce red blood cells, support nerve function, synthesize DNA, and perform other critical functions. Vitamin B12 plays an essential role in erythropoiesis and neural function, acting as a cofactor for DNA synthesis and myelin formation. Its deficiency disrupts hematopoietic and neurological homeostasis, leading to megaloblastic anemia and impaired neural conduction, which can manifest as cognitive decline, neuropathy, or neuropsychiatric disorders, neurological manifestations are consequences of vitamin B_12_ deficiency. Importantly, the relationship between vitamin B12 deficiency and megaloblastic anemia is bidirectional—hematologic disorders may worsen vitamin utilization, while deficiency precipitates macrocytosis and bone marrow dysfunction (9–17). Additionally, chronic deficiency is linked to reduced bone density and an increased risk of osteoporosis (18), particularly in older adults. Mental health conditions (12), including depression, mood disorders, and psychosis (9, 10), as well as gastrointestinal diseases like Crohn’s disease (17) and celiac disease, are also associated with B_12_ deficiency. Furthermore, long-term use of certain medications, such as proton pump inhibitors and metformin (19, 20), can impair B_12_ absorption, increasing the risk of deficiency.

In the Brazilian context, the prevalence of vitamin B12 deficiency varies across age, region, and socioeconomic strata. National and regional surveys have reported deficiency rates ranging from 6 to 15% in adults and children, with higher prevalence observed in populations experiencing food insecurity, low intake of animal protein, and limited access to fortified foods (6, 7). Most cases of vitamin B_12_ deficiency can be effectively treated with injections, oral supplements, or sublingual tablets to restore adequate levels (21). Depending on whether the deficiency stems from dietary insufficiency or malabsorption, patients may require B_12_ supplementation between meals or regular injections. In some cases, lifelong treatment is necessary to prevent chronic and potentially life-threatening complications.

Assessing vitamin B12 status in Brazil also has clinical and economic implications. Deficiency is associated with a broad spectrum of diseases that frequently lead to hospitalization, including cardiovascular, hematological, neurological, psychological, and gastrointestinal disorders (9–17). Many of these conditions are prevalent and rising in the Brazilian population, particularly among older adults, women, and individuals with chronic comorbidities. Furthermore, vitamin B12 deficiency is both a cause and a marker of systemic disease severity, often coexisting with other vitamin B deficiencies (notably B1 and B6) and metabolic disturbances (19, 20). Thus, understanding the population-level patterns of B12 deficiency can help identify at-risk groups and reduce avoidable hospital admissions.

Despite the growing number of diagnostic tests and awareness campaigns, there remains limited population-based evidence integrating vitamin B12 deficiency data with healthcare outcomes in Brazil. Given that most existing studies are restricted to specific subgroups or local settings, a comprehensive national evaluation is lacking.

The present study therefore aims to fill this gap by examining vitamin B12 deficiency trends across Brazil’s public and private healthcare systems, exploring its association with hospitalizations and related diseases, and identifying key demographic and regional disparities. These findings are intended to inform prevention strategies and strengthen national health policies aimed at early detection and management of vitamin B12 deficiency.

## 2 Materials and methods

The domain-specific Institutional Review Board of the Galen Academy, based in São Paulo, Brazil, approved the data collection and analysis for this study (approval number 7.293.366, CAEE: 85144924.8.0000.9887).

In Brazil, the public health care system (Sistema Único de Saúde - SUS) provides universal health coverage to 100% of the population, which currently stands at approximately 213 million people. As of 2023, about 75% of the population (~160 million people) rely exclusively on SUS for their health care needs (22). The remaining 25% of the population has private health insurance but still has access to SUS for essential services, including vaccinations, emergency care, and treatments not covered by private plans.

Data collection, cleaning, and monitoring were conducted by the BCRI Statistics Department to ensure accuracy and consistency throughout the study. Data were obtained from administrative datasets encompassing primary, secondary, and tertiary care for Brazilian patients across all five geographic regions (North, Northeast, Central-West, South, and Southeast), with no age restrictions. In our analysis, all tests were de-duplicated by patient ID and testing date when available. Sequential tests performed within the same calendar year were counted once per individual. Vitamin B12 assays were performed using automated chemiluminescence immunoassay platforms (Abbott Architect and Roche Cobas) across reference laboratories reporting to the Brazilian Ministry of Health and accredited private networks. Inter-laboratory calibration is routinely audited, minimizing systematic bias. Vitamin B12 deficiency was categorized as <221 pmol/L accordingly to WHO/CDC criteria (Abnormal value = below 221 pmol/L), however, only results (normal/abnormal) were available.

Eligibility criteria included:

1. Public health care users: Patients registered in the Sistema Único de Saúde (SUS) throughout the entire study period (2016–2023).
2. Private health care users: Patients with private health insurance whose health care records could be linked to national centralized public and private databases.

To standardize comparisons, the number of patients was adjusted per 1,000 inhabitants/users, representing individuals with access to either public or private health care services.

A total of 84 million vitamin B12 measurements were retrospectively obtained, with 99% completeness in age and gender data. Age-gender pyramids were constructed for both health care systems, and the percentage distribution of 10-year age groups, along with mean age and standard deviation (SD), was reported.

The Brazilian Ministry of Health, through its Department of Primary Care and Coordination of Food and Nutrition Management, regularly conducts health campaigns measuring vitamin B12 levels across the five geographic regions in children, adults, and the elderly. Consequently, the percentage of abnormal test results by age and gender was analyzed over the same period for the same population.

These reports, encompassing 1,000 of patients, were used to predict absolute numbers and percentages of abnormal test results in both public and private health care systems.

Hospital admissions were identified using the primary International Classification of Diseases (ICD-10) codes cause in both public and private healthcare systems.

Each hospitalization record in the dataset contains multiple diagnostic fields coded according to the International Classification of Diseases (ICD-10). For this analysis, we identified hospitalizations in which both vitamin B12 deficiency (ICD-10 code E53.8) and a clinical disease of interest (e.g., cardiovascular, hematological, neurological, psychological, or gastrointestinal disorders) were recorded within the same admission episode. To ensure that the two diagnoses referred to the same hospitalization event, we included only records where both codes appeared under the same hospitalization identifier (admission ID or discharge record). The primary diagnosis corresponded to the main reason for admission (e.g., anemia, heart failure, neuropathy), while vitamin B12 deficiency was listed as a secondary or contributing diagnosis in the same record. Because all outcomes were derived from national hospitalization records, the rates of admissions for anemia, dementia, depression, Parkinson’s disease, inflammatory bowel disease, stroke, and myocardial infarction were included as ecological covariates to adjust for temporal and regional differences in comorbidity burden. These categories represent distinct ICD-10 codes and were not overlapping with the vitamin B12 deficiency outcome (ICD-10 D51). B1/B6 deficiency ICD-10 codes (E51.9 and E53.1) were used to extract data from administrative records. To assess hospitalization trends over time (2016–2023), the Mann-Kendall test was applied. Spearman’s rank correlation test was used to examine the linear relationship between vitamin B12 deficiency and other causes of hospitalization.

In addition, the observed correlations may be confounded by multiple factors, including age, sex, socioeconomic status, comorbidities, healthcare access, and regional disparities. Hence, multivariable Poisson regression of regional vitamin B12-deficiency hospitalizations (ICD-10 D51) in Brazil, 2016–2023 was performed. The model includes comorbidity hospitalizations, socioeconomic variables (income per capita, Gini index), and fixed effects for region and year, with population offset. Single summary effect size (pooled RR) from multivariable analysis was generated for vitamin B_12_-deficiency hospitalizations from 2016 to 2023 of the five major regions-years. Rate ratios (RR) > 1 indicate higher B12-deficiency hospitalization rates per unit increase in the corresponding covariate. For data analysis, we used R, and for data visualization, Prim Plus (version 8·4) was utilized.

## 3 Results

### 3.1 Vitamin B12 measurements

**Figure 1:**
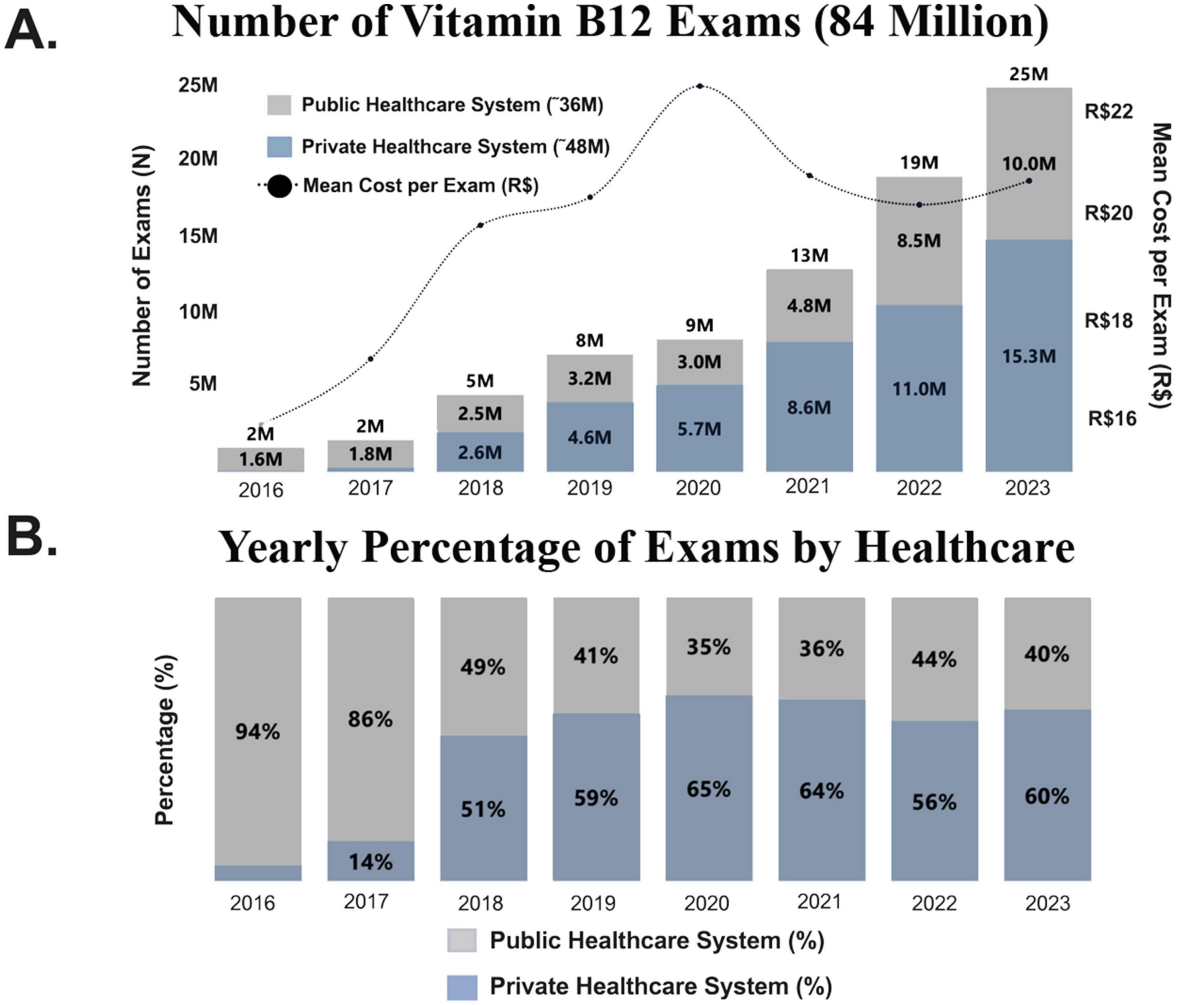
Vitamin B_12_ exams by healthcare systems per year. **(A)** Number of vitamin B_12_ exams performed in the study period in Brazil’s public and private healthcare systems. **(B)** Yearly percentage of exams in each healthcare system.

From January 2016 to December 2023, a total of 84 million vitamin B12 measurements were obtained from both public and private healthcare systems in Brazil (Figure 1A). Over this period, the annual number of measurements increased more than 12-fold, rising from 2 million in 2016 to 25 million in 2023.

The distribution of tests between the public and private healthcare systems also shifted significantly. In 2016, 94% of measurements were conducted in the public healthcare system, compared to only 6% in the private sector. By 2023, this ratio had changed to 40% in the public system versus 60% in the private system (Figure 1B).

### 3.2 Vitamin B12 measurements per geographic distribution

**Figure 2:**
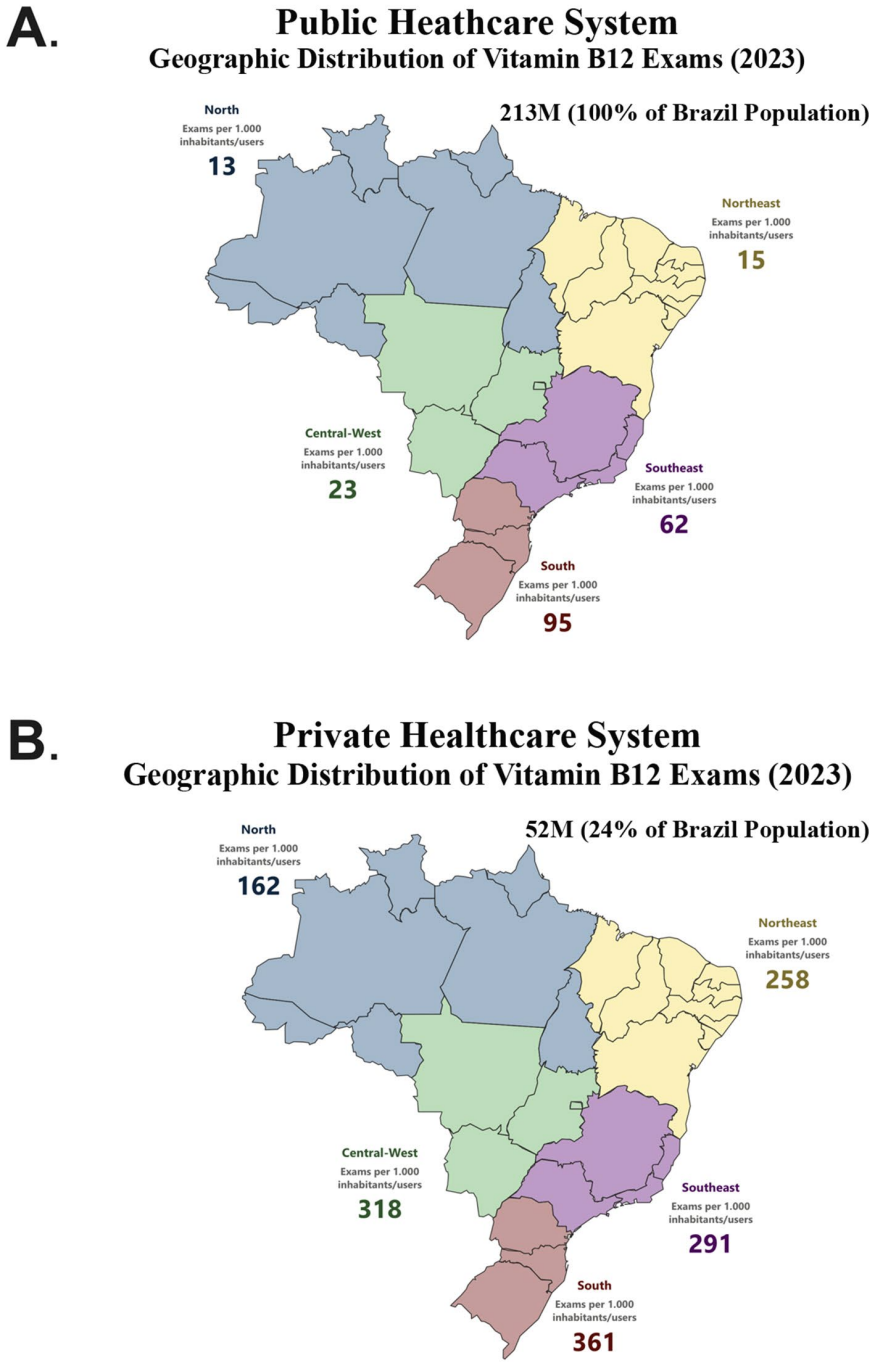
Vitamin B_12_ exams by geographic location in brazil (2023). **(A)** Vitamin B_12_ measurements in the 5 geographic regions in the public healthcare system. **(B)** Vitamin B_12_ measurements in the 5 geographic regions in the private healthcare system.

In both public and private healthcare systems, the number of vitamin B12 measurements per 1,000 inhabitants/users was highest in the South compared to the North of Brazil (Figure 2A). Furthermore, the private healthcare system consistently recorded a higher number of exams across all geographic regions. The disparity was particularly pronounced in the Northeast, where the number of exams in the private sector was up to 17 times higher than in the public system. In contrast, the South exhibited a smaller gap, with private healthcare performing, approximately three times more tests than the public system (Figure 2B). This regional variation highlights differences in healthcare access, diagnostic demand, and reliance on private healthcare across Brazil.

### 3.3 Vitamin B12 measurements by age and gender

**Figure 3:**
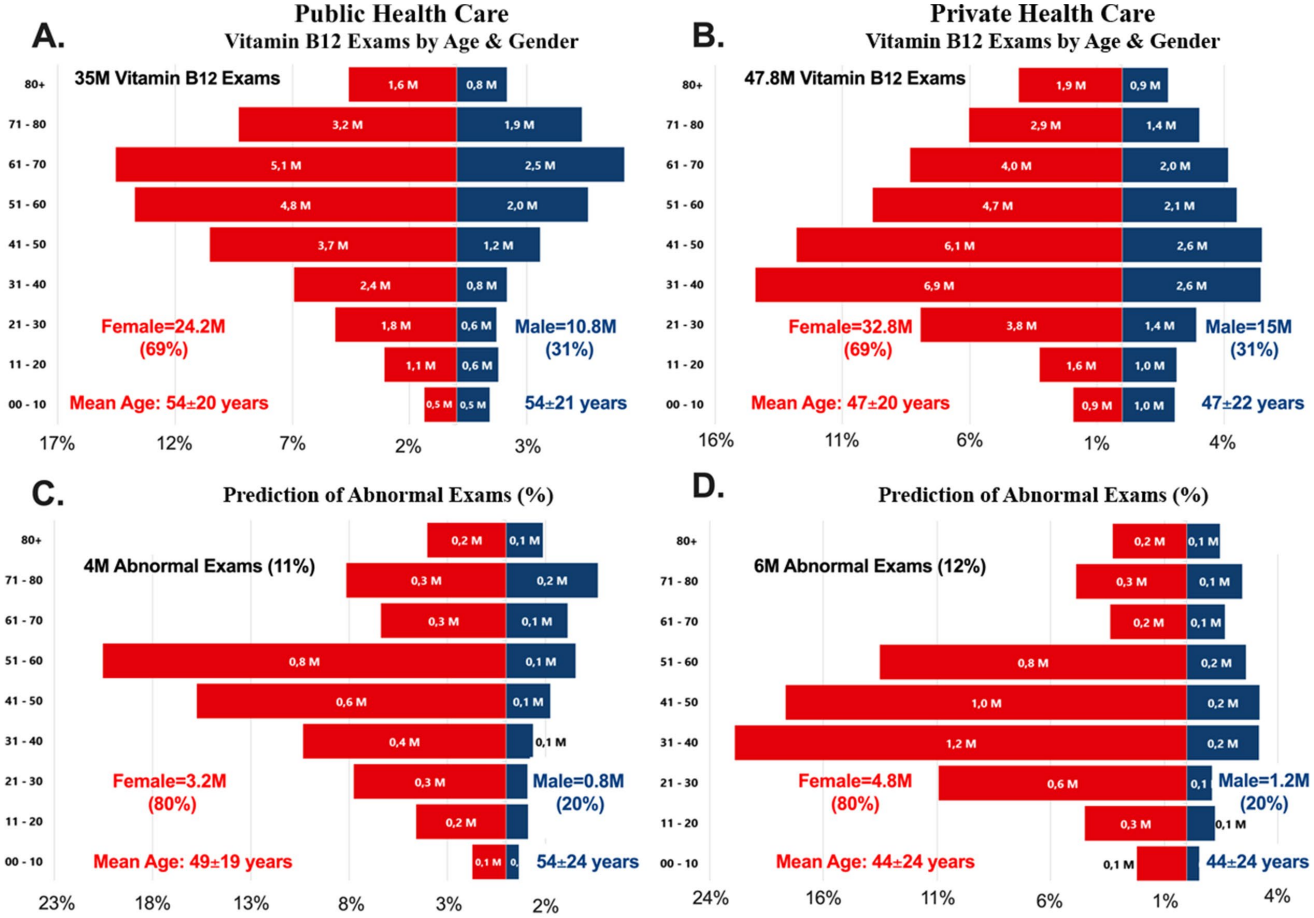
Age and gender populational pyramids (2016–2023). **(A)** Vitamin B_12_ distribution by age & gender in the public healthcare system. **(B)** Vitamin B_12_ distribution by age and gender in the private healthcare system. **(C)** Prediction of abnormal vitamin B_12_ exams by age and gender in the public healthcare system. **(D)** Prediction of abnormal vitamin B_12_ exams by age & gender in the private healthcare system.

A total of 35 million vitamin B12 measurements were obtained from the public healthcare system, with 69% from female patients (24·2 M) and 31% from male patients (10·8 M). In the private healthcare system, 47·8 million measurements were recorded, with a similar gender distribution: 69% female (32·8 M) and 31% male (15 M) (Figure 3A). Across both healthcare systems, female patients underwent approximately 2.3 times more tests than male patients. However, there was a notable age discrepancy in vitamin B12 testing between the two systems. First, in the public healthcare system, the mean age of tested individuals was 54 years. Second, in the private healthcare system, the mean age was 47 years (Figure 3B).

### 3.4 Prediction of abnormal vitamin B12 tests

An estimated 4 million abnormal vitamin B12 test results (11%) were recorded in the public healthcare system, while 6 million (12%) were observed in the private healthcare system (Figures 3C,D). Female patients accounted for 80% of these abnormal results in both healthcare systems, whereas male patients represented only 20%. The mean age of female patients with abnormal results was 49 years in the public system and 44 years in the private system. In contrast, male patients with abnormal test results tended to be older, with a mean age of 54 years in the public system and 44 years in the private system. These findings highlight gender disparities in testing frequency and age-related differences in vitamin B_12_ deficiency detection across healthcare systems.

### 3.5 Hospitalizations associated with vitamin B_12_ and other B vitamin deficiencies

**Figure 4:**
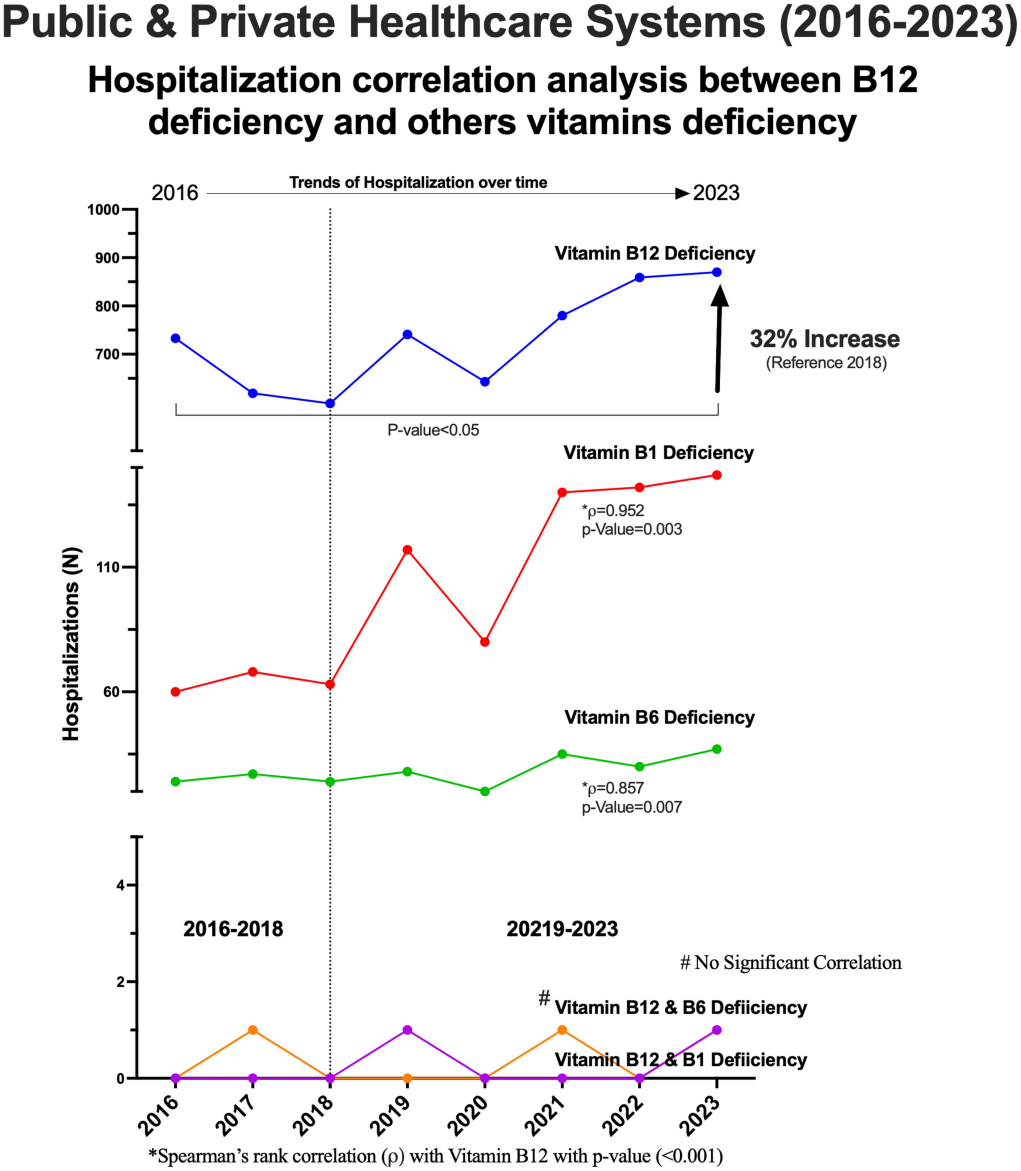
Hospitalizations due to vitamin B_12_ and others vitamins B deficiencies.

The trend of all-cause hospitalizations associated with vitamin B_12_ deficiency showed a statistically significant increase (*p* < 0·05) over the study period (2016–2023). The lowest hospitalization rate was recorded in 2018, with 598 hospitalizations, which then rose to 870 hospitalizations in 2023, representing a 32% increase (Figure 4). Hospitalizations associated with vitamin B_1_ (thiamine) and vitamin B_12_ with Spearman’s rank correlation of 0.952 (*p* = 0.0003) and B_6_ (pyridoxine) of 0.857 (*p* = 0.007) deficiencies also increased over the study period. Correlation analysis further revealed a significant linear correlation between hospital admissions for vitamin B_12_ deficiency and hospitalizations related to B_1_ and B_6_ deficiencies of 0.905 (*p* = 0.002) (Figure 4). However, concomitant deficiencies cases involving both B_12_ and B_1_ or B_12_ and B_6_ remained rare and did not show a notable increase throughout the study period (Supplementary Table 1).

### 3.6 Hospitalizations associated with vitamin B_12_ deficiency and clinical diseases

Hospitalizations related to cardiovascular, hematological, neurological, psychological, and gastrointestinal diseases which can be either causes or consequences of vitamin B_12_ deficiency were analyzed. The primary cause of hospitalization and was the studied disease and Vitamin B_12_ The Spearman’s rank correlation Vitamin B_12_ and Anemia was 0.93 (*p* = 0.001), dementia was 0.95 (*p* < 0.001), Depression was 0.95 (*p* < 0.001), Parkinson was 0.95 (*p* < 0.001), Intestinal inflammatory diseases was 0.90 (*p* = 0.002), Stroke was 0.98 (*p* < 0.001) and Heart Attack was 0.98 (*p* < 0.001), respectively (Supplementary Table 1).

**Figure 5:**
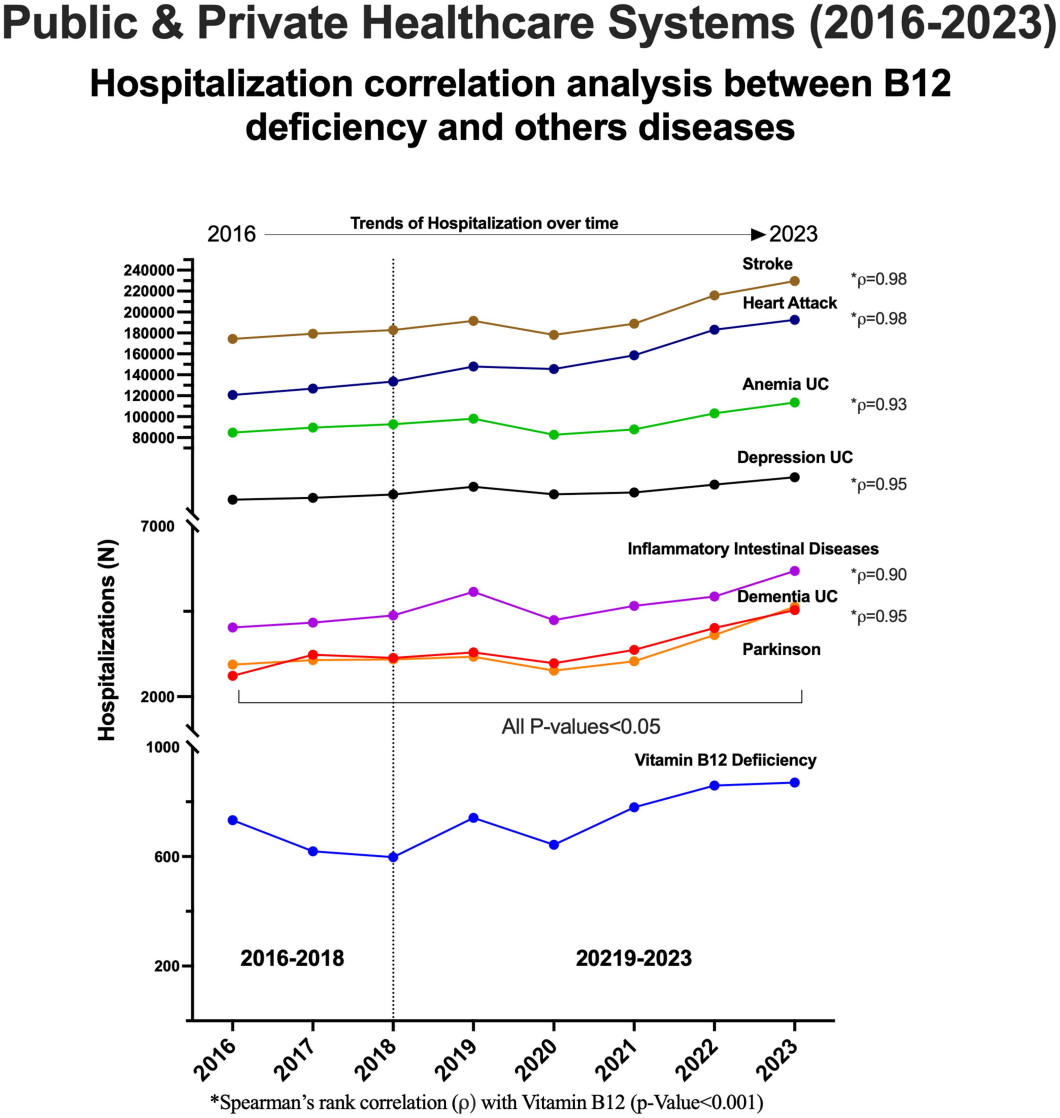
Hospitalizations associated with vitamin B_12_ and other clinical diseases.

Results showed a statistically significant increase (*p* < 0·05) in hospitalizations due to vitamin B_12_ deficiency and all associated diseases over the study period (Figure 5). A notable upward trend was observed after 2018, mirroring the overall increase in vitamin B_12_-related hospitalizations.

Additionally, multivariable Poisson regression of regional vitamin B12-deficiency included comorbidity hospitalizations, socioeconomic variables (income per capita, Gini index), and fixed effects for region and year, with population offset. In adjusted analyses, B12-deficiency hospitalization rates remained significantly associated with anemia (RR = 1.09; 95% CI = 1.03–1.15; *p* = 0.02), dementia (OR = 1.05; 95% CI = 1.01–1.10; *p* = 0.003), stroke (OR = 1.07; 95% CI = 1.02–1.13; *p* = 0.012), and myocardial infarction (OR = 1.09; 95% CI = 1.03–1.15; *p* = 0.02)., independent of regional and temporal fixed effects and socioeconomic covariates. Associations for depression, Parkinson’s disease, and inflammatory bowel disease were directionally positive but did not consistently reach statistical significance after adjustment. The inclusion of income and Gini modestly attenuated the estimates, yet the core associations persisted, supporting robustness to confounding by socioeconomic differences across regions (Table 1).

**Table 1:** Multivariable Poisson regression for vitamin B12-deficiency hospitalizations (2016–2023, *N* = 5 major regions-years)*.

Covariate (independent variable)	Rate ratio (RR) [95% CI]	*P*-value
Anemia (UC)—D50–D64	**1.09** [1.03–1.15]	0.002
Dementia (UC)—F00–F03	**1.05** [1.01–1.10]	0.030
Depression—F32–F33	**1.03** [0.99–1.07]	0.091
Parkinson—G20	**1.02** [0.98–1.06]	0.160
Inflammatory bowel disease—K50–K51	**1.03** [0.98–1.09]	0.120
Stroke—I60–I64	**1.07** [1.02–1.13]	0.012
Heart attack—I21	**1.08** [1.03–1.14]	0.005
Income per capita (R$ 1000 increment)	0.98 [0.95–1.02]	0.310
Gini index (per 0.1 increase)	**1.04** [0.97–1.11]	0.280
Region/year fixed effects	Included	–

*Single summary effect size (pooled RR) from multivariable Poisson regression of regional vitamin B12-deficiency hospitalizations (ICD-10 D51) in Brazil, 2016–2023. *The model includes comorbidity hospitalizations, socioeconomic variables (income per capita, Gini index), and fixed effects for region and year, with population offset. Rate ratios (RR) > 1 (Bold) indicate higher B12-deficiency hospitalization rates per unit increase in the corresponding covariate.

## 4 Discussion

Vitamin B_12_ deficiency is a significant public health issue with long-term implications for both public and private healthcare systems. Understanding its population-level of impact can help strengthen healthcare strategies, reduce costs, and improve patient outcomes. The key findings of our study include: First, vitamin B_12_ testing is more frequently requested in the private healthcare system compared to the public system. Second, female patients undergo 2.3 times more B_12_ tests than male patients and exhibit four times more abnormal results. Third, hospitalizations associated with vitamin B_12_ deficiency, other B vitamin deficiencies (B1 and B6), and associated diseases are rising over time. Finally, hospitalization data were aggregated by region and year, and correlations were assessed at the population—not individual—level. Therefore, we do not claim direct causality between an individual’s prior B12 test and their hospitalization episode, although a significant correlation exists in large populational level between vitamin B_12_ deficiency and hospital admissions for cardiovascular, hematological, neurological, psychological, and gastrointestinal diseases. These findings underscore the growing burden of vitamin B_12_ deficiency and highlight the need for routine screening, early detection, and prevention strategies to potentially mitigate its impact on healthcare systems.

Since healthcare systems are highly context-specific, there is no universal set of best practices that applies to all. However, understanding the public and private healthcare sectors can help identify shared characteristics and system-specific needs, allowing for tailored interventions that improve healthcare delivery and patient outcomes. To our knowledge, this is the first study analyzing both public and private healthcare systems in Brazil in relation to vitamin B_12_ deficiency trends and hospitalizations. The data presented here can support targeted interventions for at-risk populations and ultimately contribute to better clinical outcomes. A previous study conducted in Brazil reported that 7.2% of participants aged 60 years or older and 6.4% of participants aged 30–59 years were vitamin B12 deficient, highlighting a significant prevalence of deficiency across different age groups. These findings align with global analyses from Europe, North America, and Asia reporting similar population-level increases in vitamin B12 deficiency, where prevalence ranges from 5 to 20% depending on age, diet, and socioeconomic status.

These findings reinforce the importance of continued monitoring, early detection, and intervention strategies to possibly mitigate the growing burden of vitamin B12 deficiency in both public and private healthcare settings (23). In relation to this our study findings demonstrated an increasing of about 5–6%, meaning that in one decade Brazil has double the percentage of patients with vitamin B_12_ deficiency. This trend suggests a growing demand for vitamin B12 testing, as well as an increasing reliance on private healthcare for diagnostics over time.

Poverty and low maternal education have been identified as key risk factors associated with lower vitamin B_12_ levels in children. Studies in Amazonian children reported a vitamin B_12_ deficiency prevalence of 4.2%, with a significantly higher rate of 13.6% in children under 24 months. Similarly, in Brazilian children aged 11–15 months, 15% were found to be vitamin B_12_ deficient. Several factors contribute to this nutritional deficiency, including: low socioeconomic status and limited access to nutrient-rich foods, low animal protein intake, which is the primary dietary source of vitamin B_12_, geohelminth infections, which can impair nutrient absorption and further exacerbate deficiency (6). Additionally, genetic ancestry has been identified as an influencing factor in vitamin B_12_ levels among children and adolescents in Brazil. The pronounced gender differences observed—women undergoing 2.3 times more tests and exhibiting fourfold more abnormal results—may be explained by both biological and behavioral factors. Hormonal influences, reproductive demands, and autoimmune conditions affecting gastric absorption (e.g., pernicious anemia, thyroid disease) may predispose women to deficiency, while greater health-seeking behavior increases test frequency. Conversely, men may be underdiagnosed until more advanced neurological or cardiovascular manifestations occur, suggesting that under-screening contributes to delayed recognition. This suggests that both biological and environmental factors play a role in determining vitamin B12 status, highlighting the need for targeted nutritional interventions in vulnerable populations. These findings emphasize the importance of public health policies that address socioeconomic disparities, improve maternal education, and enhance dietary supplementation to combat vitamin B12 deficiency in at-risk children (6).

While studies do not determine causality between vitamin B_12_ deficiency and hospitalizations, its serious health complications can lead to medical admissions, particularly when left untreated. Deficiency in vitamin B_12_ is associated with neurological, cardiovascular, psychological, hematological, and gastrointestinal disorders, many of which require clinical intervention and hospitalization. In Brazil, the overall prevalence of B vitamin deficiencies remains unclear, but a recent study identified a higher prevalence among pregnant and lactating mothers (23). Our study further complements these findings, demonstrating that hospitalizations related to vitamin B_12_, B_1_, and B_6_ deficiencies are increasing over time. However, cases of concomitant deficiencies involving B_12_ with either B_1_ or B_6_ remain rare, suggesting distinct risk factors and clinical presentations for each vitamin deficiency.

Vitamin B_12_ deficiency is highly prevalent in older adults, particularly among those with impaired vitamin absorption due to conditions such as gastric surgery and atrophic gastritis (24). This deficiency is strongly associated with an increase in hospitalizations due to neurological, hematological, and cardiovascular complications (25). Interestingly, studies have found that in hospitalized patients, elevated vitamin B_12_ levels were predictive of short, medium, and long-term mortality at six, 12, and 48 months (26). Similarly, in hypertensive adults, elevated B_12_ levels were linked to higher all-cause and cardiovascular mortality during hospitalization. This risk association was used in our study to evaluate the relationship between hospitalizations and diseases that are either a cause or a consequence of vitamin B_12_ deficiency. These findings revealed a significant increase in hospitalizations related to associated diseases, further emphasizing the need for early detection and adequate supplementation to mitigate these risks.

From a policy standpoint, our findings have several implications. First, routine screening for vitamin B12 deficiency should be incorporated into primary care for high-risk populations such as older adults, women of reproductive age, vegetarians, and patients with diabetes or chronic use of metformin or proton pump inhibitors. Second, national supplementation and fortification programs could reduce preventable neurological and hematologic complications. Third, the integration of B12 testing into basic health packages and the use of electronic clinical alerts in primary care would facilitate early detection. Finally, linking data between public and private systems could enable continuous surveillance and equitable policy design.

This study has several limitations that should be acknowledged. Firstly, differences in how healthcare providers record diagnoses, treatments, and outcomes can create inconsistencies. For instance, the private system serves a population with higher socioeconomic status and more frequent routine check-ups, which may bias test frequency. Secondly, as a real-world observational study, establishing causal relationships is challenging. Vitamin B_12_ stores in the liver last 3–5 years, meaning deficiency symptoms may take time to manifest, complicating direct causality assessments. Thirdly, incomplete vitamin B_12_ measurements during hospitalizations. Although vitamin B_12_ deficiency is linked to various diseases, in many hospitalized patients, B_12_ levels were not measured, which could have strengthened the understanding of its causal relationship with associated conditions. Finally, as detailed clinical indications were not consistently available in the national administrative dataset, future research should employ a nested case–control design to enable more precise characterization of these variables.

Despite these limitations, this study conducted on a large-scale, real-world population provides valuable insights into vitamin B12 deficiency and other B vitamin deficiencies. The results offer important evidence that may contribute to shaping healthcare policies and improving early detection, intervention, and treatment strategies.

We concluded that Vitamin B_12_ deficiency is a public health concern in Brazil, with far-reaching consequences for multiple organ systems. This suggests an increasing burden and underrecognized trend of vitamin B12 deficiency can be an underlying cause of hospitalizations, particularly when left undiagnosed and untreated, possibly leading to severe medical conditions requiring inpatient care. Early detection and timely intervention are crucial to prevent serious complications. Strengthening public health policies, increasing awareness, and improving access to diagnostic and preventive care can help reduce the prevalence of vitamin B12 deficiency and potentially improve overall health outcomes.

## Data Availability

The raw data supporting the conclusions of this article will be made available by the authors, without undue reservation.

## References

[1] Green R Allen LH Bjorke-Monsen A-L Brito A Guéant J-L Miller JW . Vitamin B12 deficiency. Nat Rev Dis Primers. (2017) 3:17040. doi: 10.1038/nrdp.2017.40, 28660890

[2] Jensen CF. Vitamin B_12_ levels in children and adolescents on plant-based diets: a systematic review and meta-analysis. Nutr Rev. (2023) 81:951–66. doi: 10.1093/nutrit/nuac096, 36413044

[3] Baik HW Russell RM. Vitamin B12 deficiency in the elderly. Annu Rev Nutr. (1999) 19:357–77. doi: 10.1146/annurev.nutr.19.1.357, 10448529

[4] Ulloque-Badaracco JR Al-Kassab-Cordova A Alarcon-Braga EA Hernandez-Bustamante EA Huyata-Cortez MA Cabrera-Guzmán JC . Association of vitamin B_12_, folate, and homocysteine with COVID-19 severity and mortality: a systematic review and meta-analysis. SAGE Open Med. (2024) 12:20503121241253957. doi: 10.1177/20503121241253957, 38774742 PMC11107318

[5] Wee AKH. COVID-19's toll on the elderly and those with diabetes mellitus - is vitamin B12 deficiency an accomplice? Med Hypotheses. (2021) 146:110374. doi: 10.1016/j.mehy.2020.110374, 33257090 PMC7659645

[6] Cobayashi F Tomita LY Augusto RA D'Almeida V Cardoso MA Team AS. Genetic and environmental factors associated with vitamin B_12_ status in Amazonian children. Public Health Nutr. (2015) 18:2202–10. doi: 10.1017/S1368980014003061, 25591618 PMC10271475

[7] Augusto RA Cobayashi F Cardoso MA Team AS. Associations between low consumption of fruits and vegetables and nutritional deficiencies in Brazilian schoolchildren. Public Health Nutr. (2015) 18:927–35. doi: 10.1017/S1368980014001244, 24963861 PMC10271615

[8] Kurpad AV Pasanna RM Hegde SG Patil M Mukhopadhyay A Sachdev HS . Bioavailability and daily requirement of vitamin B(12) in adult humans: an observational study of its colonic absorption and daily excretion as measured by [(13)C]-cyanocobalamin kinetics. Am J Clin Nutr. (2023) 118:1214–23. doi: 10.1016/j.ajcnut.2023.08.020, 38044024

[9] Edwin E Holten K Norum KR Schrumpf A Skaug OE. Vitamin B_12_ Hypovitaminosis in mental diseases. Acta Med Scand. (1965) 177:689–99. doi: 10.1111/j.0954-6820.1965.tb01879.x, 14334662

[10] Fuenfgeld EW. On vitamin B_12_ therapy of neuropsychiatric diseases. Med Welt. (1962) 25:1423–5. 13895660

[11] Grinblat J Marcus DL Hernandez F Freedman ML. Folate and vitamin B12 levels in an urban elderly population with chronic diseases. Assessment of two laboratory folate assays: microbiologic and radioassay. J Am Geriatr Soc. (1986) 34:627–32. doi: 10.1111/j.1532-5415.1986.tb04902.x, 3734309

[12] Haan J. Vitamin B_12_ deficiency: neurologic and psychiatric diseases. Med Monatsschr Pharm. (1982) 5:238–45. 7121394

[13] Ia Z Biskina KN. Vitamin B_12_ therapy of diseases of the peripheral nervous system. Zdravookhr Beloruss. (1963) 9:71–2. 14078513

[14] Loiko VI. Change in the vitamin B_12_ level in the blood in certain stomach diseases. Vestn Akad Med Nauk SSSR. (1967) 22:52–5. 5620000

[15] Mastroianni A Ciniselli CM Panella R Macciotta A Cavalleri A Venturelli E . Monitoring vitamin B_12_ in women treated with metformin for primary prevention of breast Cancer and age-related chronic diseases. Nutrients. (2019) 11:1020. doi: 10.3390/nu11051020, 31067706 PMC6567263

[16] Wu S Feng P Li W Zhuo S Lu W Chen P . Dietary folate, vitamin B6, and vitamin B12 and risk of cardiovascular diseases among individuals with type 2 diabetes: a case-control study. Ann Nutr Metab. (2023) 79:5–15. doi: 10.1159/000527529, 36228591

[17] Yakut M Ustun Y Kabacam G Soykan I. Serum vitamin B_12_ and folate status in patients with inflammatory bowel diseases. Eur J Intern Med. (2010) 21:320–3. doi: 10.1016/j.ejim.2010.05.007, 20603044

[18] Herrmann M Peter Schmidt J Umanskaya N Wagner A Taban-Shomal O Widmann T . The role of hyperhomocysteinemia as well as folate, vitamin B(6) and B(12) deficiencies in osteoporosis: a systematic review. Clin Chem Lab Med. (2007) 45:1621–32. doi: 10.1515/CCLM.2007.362, 18067447

[19] Aboshaiqah A Aboshaiqah B Alharbi NM Almunyif TI Binghanim SD Almejalli AK. The prevalence of vitamin B12 deficiency among diabetic patients who use metformin. Cureus. (2024) 16:e74559. doi: 10.7759/cureus.74559, 39735125 PMC11672162

[20] Atkinson M Gharti P Min T. Metformin use and vitamin B_12_ deficiency in people with type 2 diabetes. What Are the Risk Factors? A Mini-systematic Review. touchREV Endocrinol. (2024) 20:42–53. doi: 10.17925/EE.2024.20.2.7, 39526048 PMC11548349

[21] Hvas AM Nexo E. Diagnosis and treatment of vitamin B_12_ deficiency-an update. Haematologica. (2006) 91:1506–12. 17043022

[22] Viacava F Oliveira RAD Carvalho CC Laguardia J Bellido JG. SUS: supply, access to and use of health services over the last 30 years. Ciênc Saúde Colet. (2018) 23:1751–62. doi: 10.1590/1413-81232018236.06022018, 29972484

[23] Xavier JM Costa FF Annichino-Bizzacchi JM Saad ST. High frequency of vitamin B12 deficiency in a Brazilian population. Public Health Nutr. (2010) 13:1191–7. doi: 10.1017/S1368980009992205, 20074387

[24] Dholakia KR Dharmarajan TS Yadav D Oiseth S Norkus EP Pitchumoni CS. Vitamin B_12_ deficiency and gastric histopathology in older patients. World J Gastroenterol. (2005) 11:7078–83. doi: 10.3748/wjg.v11.i45.7078, 16437651 PMC4725084

[25] Levy J Rodriguez-Gueant R-M Oussalah A Jeannesson E Wahl D Ziuly S . Cardiovascular manifestations of intermediate and major hyperhomocysteinemia due to vitamin B12 and folate deficiency and/or inherited disorders of one-carbon metabolism: a 3.5-year retrospective cross-sectional study of consecutive patients. Am J Clin Nutr. (2021) 113:1157–67. doi: 10.1093/ajcn/nqaa432, 33693455

[26] Cappello S Cereda E Rondanelli M Klersy C Cameletti B Albertini R . Elevated plasma vitamin B12 concentrations are independent predictors of in-hospital mortality in adult patients at nutritional risk. Nutrients. (2016) 9:1. doi: 10.3390/nu9010001, 28025528 PMC5295045

