# Supplementary material for: Vitamin B12 deficiency and its impact on healthcare: a population-level analysis and call for action
Source: Front Nutr. 2026 Jan 5;12:1701661. doi: 10.3389/fnut.2025.1701661 (PMC12812575; doi:10.3389/fnut.2025.1701661)
Supplement: Supplementary file 1 [file Table_1.DOCX]

**Complementary Table 1. Correlations between B12 Vitamin**

| **Correlation analysis between B12 deficiency and other Vitamins** | | |
| --- | --- | --- |
| **Vitamins** | **ρ (rho)** | **p-value** |
| B12 vs B1 | 0.952 | 0.0003* |
| B12 vs B6 | 0.857 | 0.007* |
| B12 vs B12+B1 | 0.447 | 0.26 |
| B12 vs B12+B6 | 0.509 | 0.20 |
| B1 vs B6 | 0.905 | 0.002* |
| B1 vs B12+B1 | 0.477 | 0.23 |
| B1 vs B12+B6 | 0.509 | 0.20 |
| B6 vs B12+B1 | 0.595 | 0.13 |
| B6 vs B12+B6 | 0.647 | 0.08 |
| B12+B1 vs B12+B6 | 0.447 | 0.26 |
| **Correlation analysis between B12 deficiency and various disease categories** | | |
| **Vitamin B12 vs. Diseases** | **ρ (rho)** | **p-value** |
| B12 Def vs Anemia | 0.93 | 0.001* |
| B12 Def vs Dementia | 0.95 | < 0.001* |
| B12 Def vs Depression | 0.95 | < 0.001* |
| B12 Def vs Parkinson | 0.95 | < 0.001* |
| B12 Def vs Intestinal Inflammatory Diseases | 0.90 | 0.002* |
| B12 Def vs Stroke | 0.98 | < 0.0001* |
| B12 Def vs Heart Attack | 0.98 | < 0.0001* |
| Spearman’s rank correlation |  |  |
| **p*-values <0.05 |  |  |
